# Advanced Materials Based on Nanosized Hydroxyapatite

**DOI:** 10.3390/molecules26113190

**Published:** 2021-05-26

**Authors:** Ramón Rial, Michael González-Durruthy, Zhen Liu, Juan M. Ruso

**Affiliations:** 1 Soft Matter and Molecular Biophysics Group, Department of Applied Physics, University of Santiago de Compostela, 15782 Santiago de Compostela, Spain; ramon.rial@usc.es (R.R.); gonzalezdurruthy.furg@gmail.com (M.G.-D.); 2 Department of Physics and Engineering, Frostburg State University, Frostburg, MD 21532, USA; zliu@frostburg.edu

**Keywords:** hydroxyapatite, tissue engineering, bones, porous materials, biomaterials, regenerative medicine

## Abstract

The development of new materials based on hydroxyapatite has undergone a great evolution in recent decades due to technological advances and development of computational techniques. The focus of this review is the various attempts to improve new hydroxyapatite-based materials. First, we comment on the most used processing routes, highlighting their advantages and disadvantages. We will now focus on other routes, less common due to their specificity and/or recent development. We also include a block dedicated to the impact of computational techniques in the development of these new systems, including: QSAR, DFT, Finite Elements of Machine Learning. In the following part we focus on the most innovative applications of these materials, ranging from medicine to new disciplines such as catalysis, environment, filtration, or energy. The review concludes with an outlook for possible new research directions.

## 1. Introduction

The history of mankind is closely related to technological development. Depending on the most characteristic resources or tools, we have spoken of the stone age, bronze, iron, wood, coal, steel, petrol, and semiconductors. Nowadays, the confluence of techniques and methods from disciplines such as physics, chemistry, biology or engineering has given rise to what is known as materials science and within this discipline, and much more recently, what are known as smart materials have emerged, i.e., materials designed and manipulated to adapt and respond in a controlled manner to specific stimuli [1].

Traditionally, the development of materials has been based on the use of many elements and resources. Today, for the design of smart materials, many scientists have been inspired by the elegant solutions offered by nature. Natural materials, or biomaterials, consist of a small number of building blocks, which are therefore lighter and more abundant, to design a huge variety of structures. The key lies in a hierarchical structure, i.e., different levels of organization from basic units at the lowest level to much more complex ones at higher levels. In this way, using relatively few building blocks, a great variety of minerals, macromolecules, cells, organs and even persons have been created [2,3]. In this sense, hydroxyapatite (HAP) is an example of unity and duality, a substance that is both a mineral and a biological material. From the mineral point of view, it belongs to the apatites group, with general formula Ca_5_(PO_4_)_3_X, where, depending on the element X, they are classified as fluoropatite (F), chloropatite (Cl) and hydroxyapatite (OH). They generally crystallize within the hexagonal rhombic prism with space group P63/m and unit cell dimensions: a = 9.432 Å and c = 6.881 Å. The presence of crystalline calcium phosphate is what determines that it can crystallize into hydroxyapatite and betawhitlokite salts depending on factors such as Ca/P ratio, hydration state, impurities, and temperature. The color of the crystals can be neutral or vary from red to brown, due to iron oxide. HAP has a density of 3.16 gcm^−3^, a hardness of 5 Mohs, a melting point above 1500 °C and in nature it is found in sedimentary and metamorphic rocks. This mineral is one of the most exclusive minerals and therefore, it is not easy to find deposits anywhere in the world. From the biological point of view, hydroxyapatite and its precursors are present in different animal groups from corals or starfish to vertebrates, where it is the main inorganic constituent of bone tissues and dental enamel [4,5,6]. In living beings, it does not act alone. To fulfill its function, it works together with collagen, which is a fibrous protein of the connective tissues.

**Figure 1:**
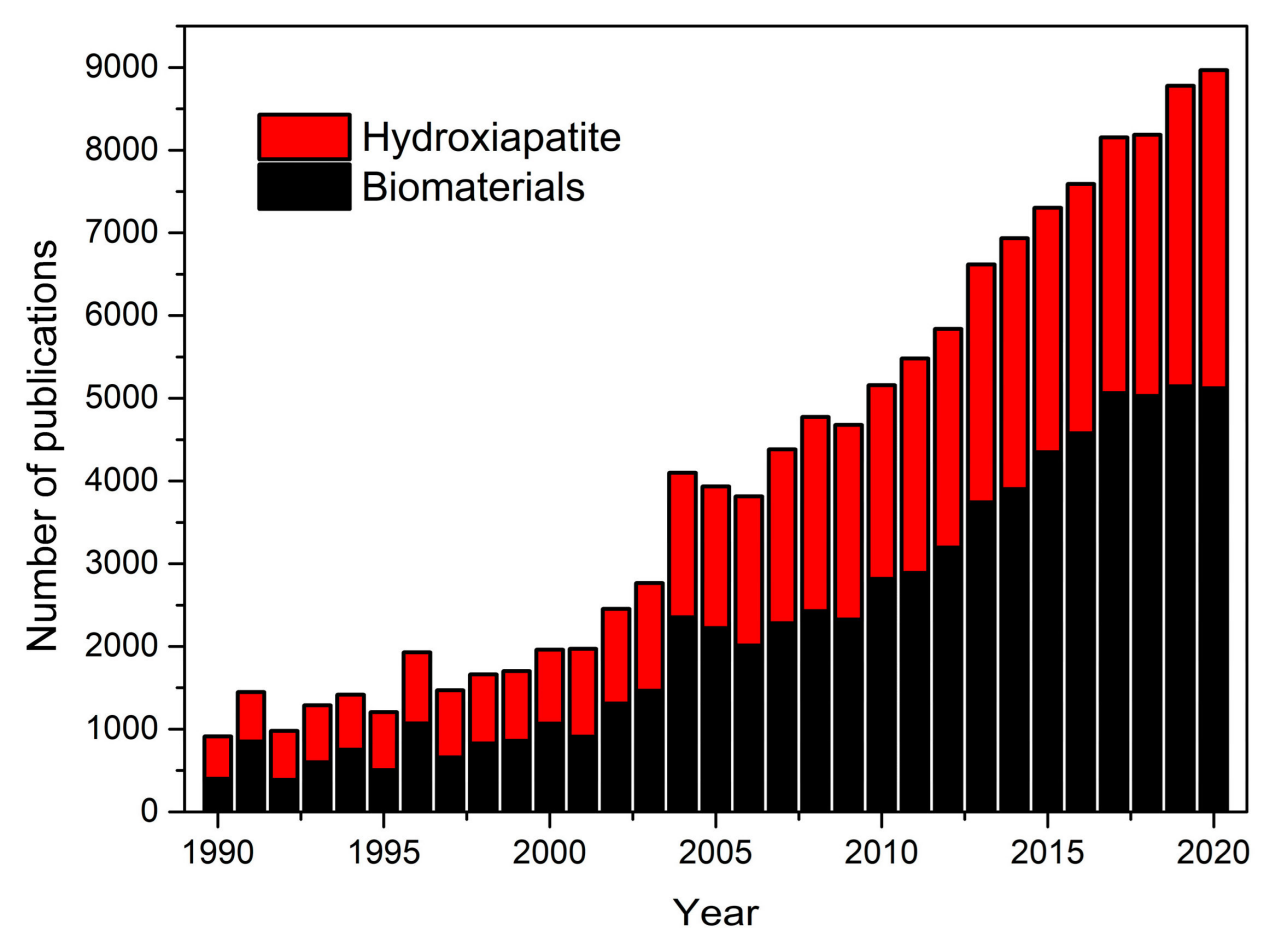
Scientific publications from 1990 to 2202 based on a Scopus database search performed in March 2021 on the topics “Biomaterials” and “Hydroxyapatite” (TITLE-ABS-KEY).

It is common to show graphs with the evolution of the number of papers published in a particular area. This type of representation does not reflect the weight that this area represents within the major scientific disciplines. In fact, as Larsen and von Ins showed, the number of publications between 1907 and 2007 has grown in all disciplines, due to the increased number of publications in all disciplines due to several factors discussed in their paper [7]. For this reason, in Figure 1, we show the number of papers where hydroxyapatite and biomaterials were employed during the last 30 years. Representing both key words, the weight of papers focused on hydroxyapatite remains intact despite the boom that the area of biomaterials has experienced in the last years [8].

In the field of biomaterials, HAP is classified as a bioactive material; that is, when it interacts with biological entities it can form interfacial bonds that, more precisely, manifest as chemical bonds between hydroxyapatite and the adjacent biological tissue. In fact, medical trials have underscored the biocompatible, bioactive, biostable and osteoconductive capabilities of hydroxyapatite [9]. It is precisely these properties that have made it one of the most widely used materials not only in those areas of medicine related to hard tissues such as: traumatology, maxillofacial or dentistry [10,11], but also in orbital implants [12]. Within these areas there it can be found either as a monolith, coatings or as one of the components of composites [13]. On the other hand, several recent studies have shown the versatility of this material, either in its pure state or combined with other elements in areas completely unrelated to tissue engineering such as: water purification, batteries, drug delivery, catalysis, ion exchange, and sensors [14,15].

**Scheme 1:**
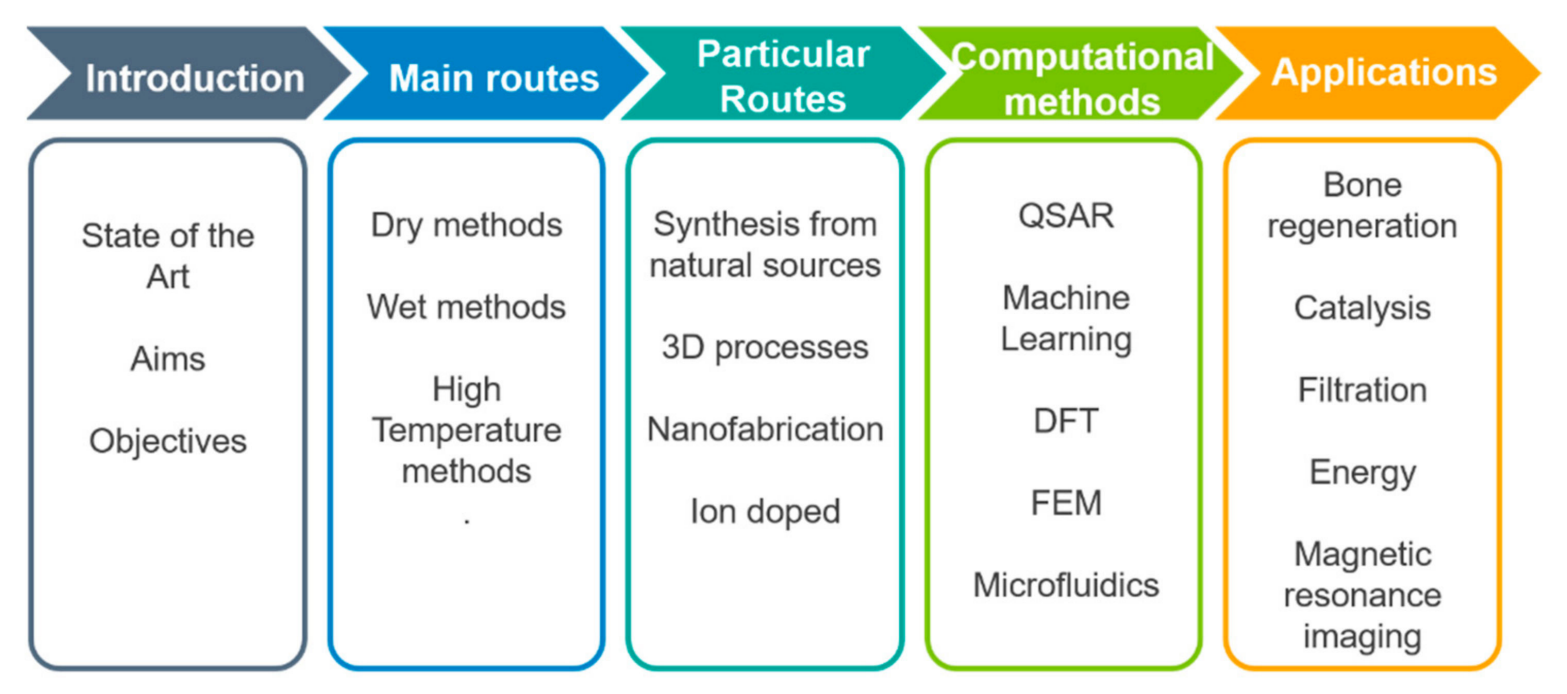
Flowchart describing the structure of this review.

Consequently, the aim of this review is to evaluate and offer a general overview about the current knowledge on hydroxyapatite based smart materials supplied by different approaches. In the compilation of this review an attempt has been made to give appropriate recognition to the current interest in most common and emergency applications, by discussing such aspects as newer systems, unusual approaches and highly used techniques including information about the physical principles and effectiveness of selected techniques (Scheme 1 depicts a flow diagram of the structure of this review). The development of nano-hydroxyapatite-based systems has raised certain misgivings; despite being chemically the same, their reduced dimensions mean that they can be easily absorbed by cells, creating adverse effects, such as cytotoxicity, induction of oxidative stress, apoptosis, and inflammatory responses. Therefore, part of this summary focuses on the evaluation of these systems. For this purpose, we have focused not only on the experimental point of view, but also in the computational one. The review is organized by techniques with the same format: beginning with a small introduction and a discussion of the specific approaches, applications and papers that are mainly based on it. It is almost impossible to cover all current techniques; therefore, we have chosen those more popular and accessible in most laboratories all over the world. Further, because of space limitations and the recent upsurge of interest in biotechnology and biomedical areas, the cited articles represent only a small fraction of the total number published in recent years.

## 2. Main Routes of Hydroxyapatite Production

In the previous section we commented that there is natural hydroxyapatite that has two major drawbacks: the presence of impurities is very high and the morphology and texture, especially at the micro scale, are predetermined. In contrast to these compounds, synthetic HAP offers the advantage of both purity and the possibility of selecting different variables such as Ca/P ratio, porosity or hardness. Thus, the number of HAP synthesis routes is large due to the different applications and the versatility of the material. There is practically a route for each specific design or application, so depending on the needs, the objectives can be to obtain HAP in powder, granular, macro- or microporous form, with different degrees of crystallinity and so on.

Although there are different classifications of synthesis routes, most authors advocate classifying them into three types: dry, wet, and high temperature processes [16,17,18,19,20,21,22]. Dry methods are sub classified in solid state and mechanochemical methods. In solid state methods, chemical precursors (such as Ca_3_(PO_4_)_2_ and CaCO_3_, CaP_2_O_7_ and CaCO_3_, or CaHPO_4_, 2H_2_O and CaO) are calcined, between 900 and 1300 °C, to obtain HAP. Basically, these processes involve the solid-state diffusion of ions from the precursors during calcination [23]. In mechanochemical processes, the chemical reaction of the precursors is induced by the action of mechanical processes such as milling or compression. The most common equipment is mixer mill and planetary mill. Rotation speed and milling time affect the obtained product: higher rotation speed increases crystallinity and crystallite size but reduces the particle size and the agglomeration of particles. On the other hand, milling time affects the HAP size and morphology. Generally speaking, dry processes ensure the formation of stoichiometric HA (Ca:P = 1.67) but require much power and time. Moreover, the products of such processes typically lack homogeneity [24,25].

The wet processes involve HAP precipitation by mixing aqueous solutions of compounds carrying Ca^2+^ and PO_4_^3−^. It is the largest group, and the different routes are classified as low-temperature chemical precipitation, hydrolysis reaction and hydrothermal method [26,27,28,29,30]. The HAP obtained by the first method usually are porous and with inhomogeneous chemical composition. The last one provides well-crystallized powders with homogeneous chemical composition [31]. In this type of synthesis, factors such as morphology, crystallinity and chemical structure show an extreme dependence on the different synthesis parameters such as reactant addition rate, concentration, drying/heat treatment conditions, and, especially, pH and temperature. The order in which the reagents are added for mixing is very important. The calcium should be added slowly to the phosphorus, otherwise the formation of pure, crystalline hydroxyapatite is hindered. The residence time plays an important role in the synthesis of pure hydroxyapatite. For short resting times, dicalcium phosphate and undefined amorphous phases may appear in addition to hydroxyapatite [32]. Sintering temperature and atmosphere are also relevant, as these factors negatively affect the mechanical strength of HAP: high sintering temperatures cause the removal of OH groups in the HAP matrix, converting part of the HAP phase into α-tricalcium phosphate, β-tricalcium phosphate and tetracalcium phosphate. In addition, this decrease in HAP phase causes the densification to disappear, negatively affecting the mechanical properties [33]. The main advantages of these methods are that they are usually the simplest and most economical routes. On the other hand, the by-product is mostly water and the probability of contamination during synthesis is very low.

Due to the great impact that HAP has on materials intended for tissue regeneration, it is important to highlight the impact of the synthesis route and methodology on the viability and final functionality of the product [34]. In this sense, powders’ strength and osteointegration are critical characteristics that depend significantly on their microstructure. Therefore, the main challenge in the synthesis of HAP for medical purposes is to control the morphological parameters that can affect its mechanical properties, biocompatibility and bioactivity [35]. In this field it is important to bear in mind that bone tissue is not uniform and has specific characteristics, for example compact or cortical bone, located on the outside, is hard and dense, and its thickness varies according to the mechanical stress; at the opposite extreme is cancellous or trabecular bone, which is generally located on the inside of the bones and is characterized by being light and having a trabecular lattice shape, with sufficient space to accommodate the marrow [36,37,38]. The great acceptance of synthetic HAP in implants or prostheses lies in its high crystallinity and the absence of carbonate, which leads to a much lower biodegradation compared to bone mineral nanocrystals. In addition, synthetic HAP facilitates the formation of chemical bonds with the host bone [39]. On the other hand, porosity control is crucial, as it provides a surface chemistry that allows bone tissue ingrowth, improving the mechanical attachment of the implant to the implantation site; in fact, if an implanted porous ceramic is progressively replaced by natural bone, its biomechanical properties become more and more similar to natural bone tissue. The importance of porosity in HAP implants has been previously documented. Minimal pore size and connectivity is required for the growth of blood vessels within the implant, a phenomenon known as osteoconduction [40,41,42,43]. Size is one of the most critical characteristics of the pores, since: pores smaller than 10 μm prevent cell access; pores from 10 to 50 μm allow penetration of fibrovascular tissue; from 50 to 150 μm allow bone penetration; pores larger than 150 μm allow bone penetration and formation [44,45]. Additionally, bone tissue penetration has been found to be possible only if pores are interconnected, but not in those that end blind [46]. Early studies conducted by Korkusov et al. [47] demonstrated that porous HAP implants are not only biomechanically stable, but in addition, there are no differences between the implanted and adjacent bone, an increase in osteoblastic activity and the stiffness in bending increased during bone healing was noted. Several authors stressed that the origin of the bioactivity of the HAP lies in the affinity of the hydroxyl groups of HAP for the amino acids, proteins and organic acids in the human body via hydrogen bonding [48]. One of the first papers documenting multistage pore formation appears in 1995, here the authors present to us the synthesis of HAP tapes made from dibasic calcium phosphate and calcium carbonate. On the basis of their experimental evidence, they conclude that the transformation of the starting powders into stoichiometric HAP occurs in two stages. The first, at about 45 °C characterized by a loss of water vapor from the calcium phosphate and a collapse of the original porosity. The second stage, at around 800 °C, involves further water loss and carbon dioxide evolution. The latter is associated with the decomposition of the calcium carbonate particles, being the responsibility of the final porous structure [49]. Klein et al. [50] succeeded in forming HAP cylinders of diameter 3.5 mm and length 7 mm, with a pore size distribution between 75 and 550 µm and between 45 and 60% porosity. Foaming methods were also employed for the synthesis of macropores in HAP structures and obtained very good pore interconnection and high porosity (60–90%) [51].

## 3. Particular Routes of Hydroxyapatite Synthesis

The idea of this subsection is to expose different alternative routes, which, although they do not have the impact of the three major routes already exposed, offer interesting opportunities that could have relevance in the immediate future. For example, biomimetic synthesis approaches can offer several advantages over conventional inorganic synthesis routes. In this sense, both new technologies and original and audacious approaches offer multiple advantages at the environmental level, in the pursuit of specific objectives or as contributions aimed at economizing certain process parameters (time, resources, energy, pollution, etc.). On the other hand, the enormous amount of organic waste generated by the food industry can offer a very interesting source of raw material.

**Figure 2:**
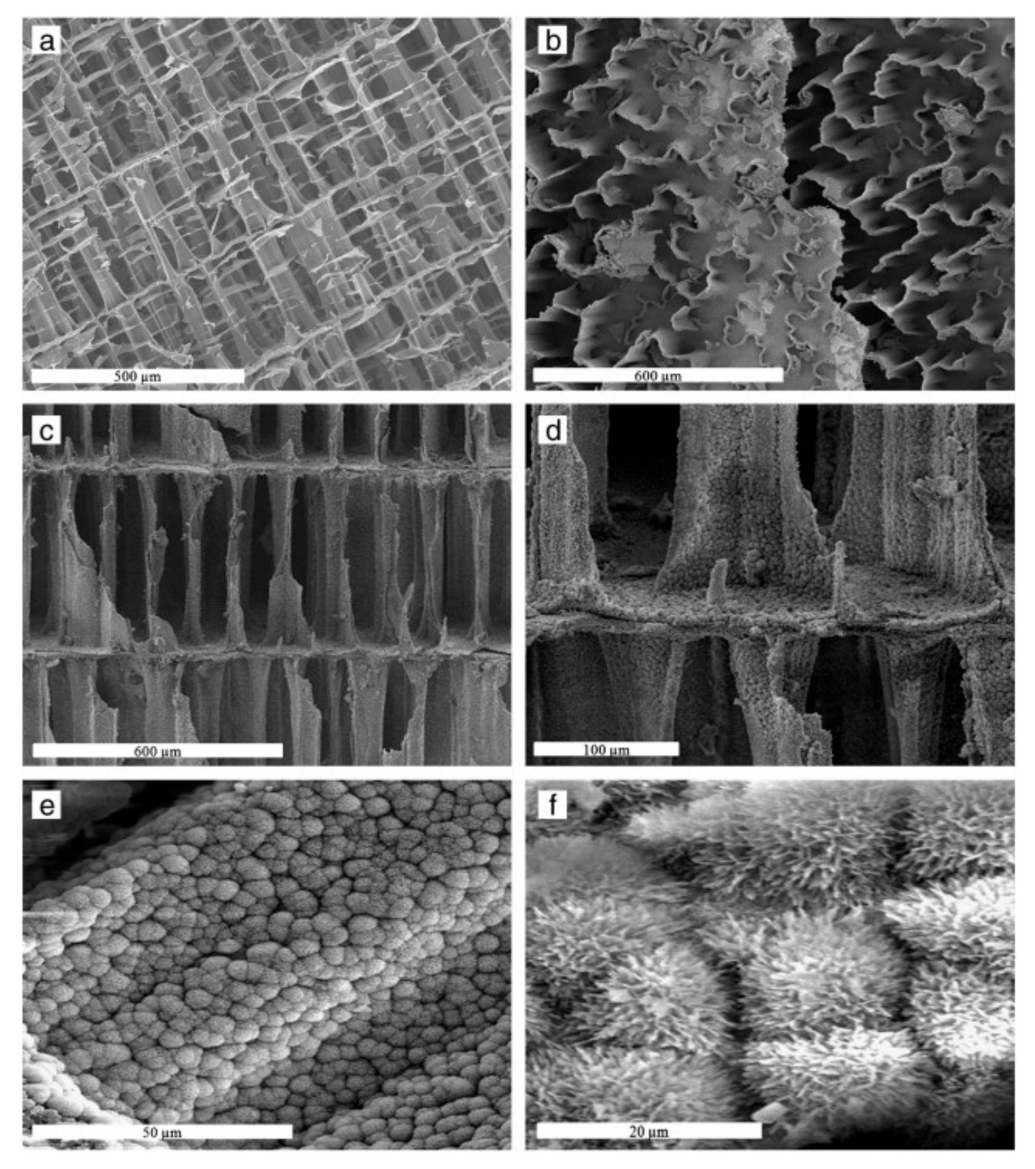
SEM images of cuttlefish bone (**a**,**b**) and cuttlefish bone after transformation into HAP (**c**–**f**). (**a**) Transverse cross-section (**b**) Pores formed by convoluted pillar (**c**,**d**) Transverse cross-section showing interconnected pores after transformation, (**e**) Aggregates of HAP and (**f**) dandelion-like structures. With permission from Elsevier https://doi.org/10.1016/j.msec.2013.09.036 (accessed on 19 May 2021).

**Figure 3:**
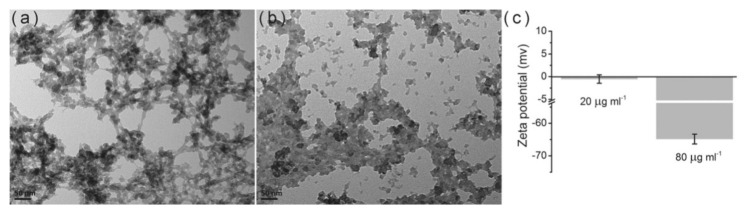
Effect of amino acid concentration on HAP particles: (**a**) low concentration; (**b**) high concentration. (**c**) zeta potential of the nanoparticles. With permission from Elsevier. https://doi.org/10.1016/j.jcrysgro.2020.125991 (accessed on 1 March 2021).

Milovac et al. [52] transformed natural aragonite from cuttlefish bone into HAP by a simple method based on hydrothermal transformation at 200 °C. The resulting product retained the cuttlebone architecture preserving the natural well interconnected channeled structure, Figure 2. This scaffold is suitable for cell attachment, proliferation and differentiation. A mechanochemical process (ball milling and heat treatment at 1000 °C) was applied to a mixture of recycled seashell and phosphoric acid to obtain high crystallinity and bioactive HAP [53]. Hen’s eggshell was the raw material to get powder-like single phase HAP in the form of globules from 4 to 5 mm, thermally stable up to 900 °C. SEM data demonstrated that the globules consist of particles with an average size of about 30 nm [54]. The same source, eggshell, was also successfully used to synthesize a porous nanohydroxyapatite/collagen composite scaffold similar to the extracellular matrices of bone and cartilage tissues. For this purpose, HAP was nucleated in the collagen matrix. The final size of the HAP was 10 nm, polycrystalline in nature and perfectly integrated into the scaffold. The scaffold showed high porosity (95–98%) with high interconnection. In addition, the mechanical strength of the scaffold doubled with the incorporation of HAP [55]. Smooth egg-shell membranes were used as a support for the formation of hydroxyapatite agglomerates in the form of a flower. The study, experimental and theoretical, shows the driving force of ions, the effect of temperature, pH and time on the morphology and crystallinity of the aggregates. These results provide important insights into the process of bone formation [56]. A combination of natural bone and shrimp shell was employed as a scaffold to be magnetically doped with Fe_3_O_4_ and Fe_2_O_3_ nanoparticle. Although the presence of the nanoparticles reduced the porosity of the matrix, the composite showed a magnetization of 3.04 emu/g [57]. Bovine cortical bone was used after heat treatment in air at 800 °C and milling to obtain HAP, then, proportional amounts of HAP and magnetic nanoparticles (Fe_3_O_4_) were compressed at 50 MPa to create the pellets. The presence of the magnetic nanoparticles in the scaffolds enhanced cell proliferation compared to pure hydroxyapatite scaffolds [58]. Ding et al. reported a facile microwave-assisted hydrothermal preparation of amorphous calcium phosphate porous hollow microspheres using adenosine triphosphate disodium salt (Na2ATP), CaCl2 and soybean lecithin in aqueous solution. This route allows modifying morphology and structure by adjusting temperature and concentrations. The hollow microspheres are suitable for drug delivery purposes [59]. Pectin, obtained from the peel of prickly pear was used as scaffold to produce, in a simple and green method, uniform HAP nanoparticles of around 25 nm. with improved antimicrobial activity towards the test pathogenic *S. aureus*, *E. coli* bacterial strains and *C. albicans* [60]. Interesting work, aimed at understanding the effect of amino acids on bone formation, was carried out by Wang et al. [61]. The authors use glutamic acid and phosphoserine to modify the structure of HAP nanocrystals. The crystals obtained without amino acids were needle-like, while crystals synthesized in the presence of the amino acids presented a platy morphology with a preferred crystal orientation on (300) face, indicating preferential adsorption and suppression of growth in specific crystal directions. The adsorption of the amino acids on the HAP surface was analyzed by molecular dynamic simulations and the results exposed that amino acids may act as effective regulators in bone biomineralization. In this same line, the dual role of acidic amino acids from non-collagenous proteins was also demonstrated [62]. The amount and place of absorption of the amino acid on the materials surface rules the aggregation evens by promoting HAP crystallization at high concentrations and inhibiting it at low concentrations. In addition, they can also change the morphology of the material by creating chain-like (Figure 3a) or liquid-like aggregates (Figure 3b).

Hutchens et al. [56] demonstrated that bacterial cellulose provides a template for the formation of biocompatible HAP spherical clusters, formed from an octacalcium phosphate precursor similar to physiological bone, with anisotropic 10–50 nm crystals elongated in the c-axis mimicking the geometry of bone apatite crystals. Different bile salts mixed aggregates were used as templates of spongy-silica materials in an approach to simulate the trabecular bone organization, from which under specific conditions the resulting materials showed an open bioactive macropore structure [63]. Nie et al. incorporated HAP into a poly(lactic-co-glycolic acid) (PLGA) matrix to prepare HAP/PLGA composites with improved cell adhesion on the composites [64]. Cui et al. prepared (polyD,L-lactic acid) PDLLA/HAP composites through the in situ growth of HAP in PDLLA, and a stable interfacial bonding was formed between them, leading to an improved tensile strength and Young’s modulus [65]. Additive manufacturing techniques provide flexible and precise controls over the size and complex shape through a layer-by-layer process but the density and mechanical properties with the printed base materials are often rare. In a recent study, the authors were able to remedy this shortcoming by using digital light processing three dimensional printing technology with mechanical results very close to the best obtained from conventional ceramic processing and sintering [66].

Li et al. [67] developed a method to construct porous hydroxyapatite by dual-phase mixing, in which the porous ceramic body and the pore-forming template are generated simultaneously. For this, the authors used a mixture of two immiscible phases: HAP slurry and polymethylmethacrylate resin. The mixture obtained is molded and subjected to polymerization, drying, pyrolysis and sintering. By controlling parameters such as viscosity, concentration ratio or mixing time and speed, it is possible to adjust porosity, pore size and interconnectivity. The material obtained was tested and proved to be sufficiently resistant for cell culture and implant handling.

Most of the production processes for 3D scaffolds are based on high temperature sintering of HAP nanoparticles for several hours. However, these processes can modify the physicochemical and biological properties of the nanoparticles, losing similarity to bone tissue. To avoid these alterations, an alternative process has been proposed in which a sodium silicate solution is used as a mineral binder for the sinterization of HAP nanoparticles by a dehydration-drying process at a low temperature (37 °C). The analysis of the final product demonstrated the viability of the new route, since from a nanoscale point of view it preserves the low crystallinity, Ca/P ratio and original size of the nanoparticles, key aspects in materials for bone regeneration. Moreover, from the microscale, the 3D scaffold has an adequate porosity and mechanical profile [68]. Silicon was also an important component of the comprehensive and rigorous study on the effect of type and chemistry of the fuel in the solution combustion synthesis process on the final properties of silicon-doped calcium phosphates carried by Farzad et al. [69]. The use of glycine as a fuel results in a higher enthalpy of combustion and concentration of gaseous by-products. The silicon-doped samples showed cytocompatibility and osteogenic potential. While the glycine and silicon mixture showed the best biological performance and the best structural and physicochemical properties.

Cyclohexane, and mixed poly (oxyethylene), nonylphenol ether were the three basic components used by Lim and his collaborators [70] in a now classic work to form a stable microemulsion to produce hydroxyapatite particles with a higher sintered density and that are more refined in grain size than that of the emulsion-derived synthesis. By optimizing the values of calcination temperature, pH and amount of components, several authors obtained a substantial improvement of the traditional Rathje method [71]. Thanks to this progress, a complete dissociation of the precursors and a refined microstructure were achieved, confirming the possibility of extending the natural precursors range for HAP preparation in an ecofriendly and cost-efficient source for bioceramics for medical applications [72]. The cytocompatibility, osteoconductivity and osteoinductivity of the materials obtained by this new route was also evaluated by the authors, although in a different article [73].

**Figure 4:**
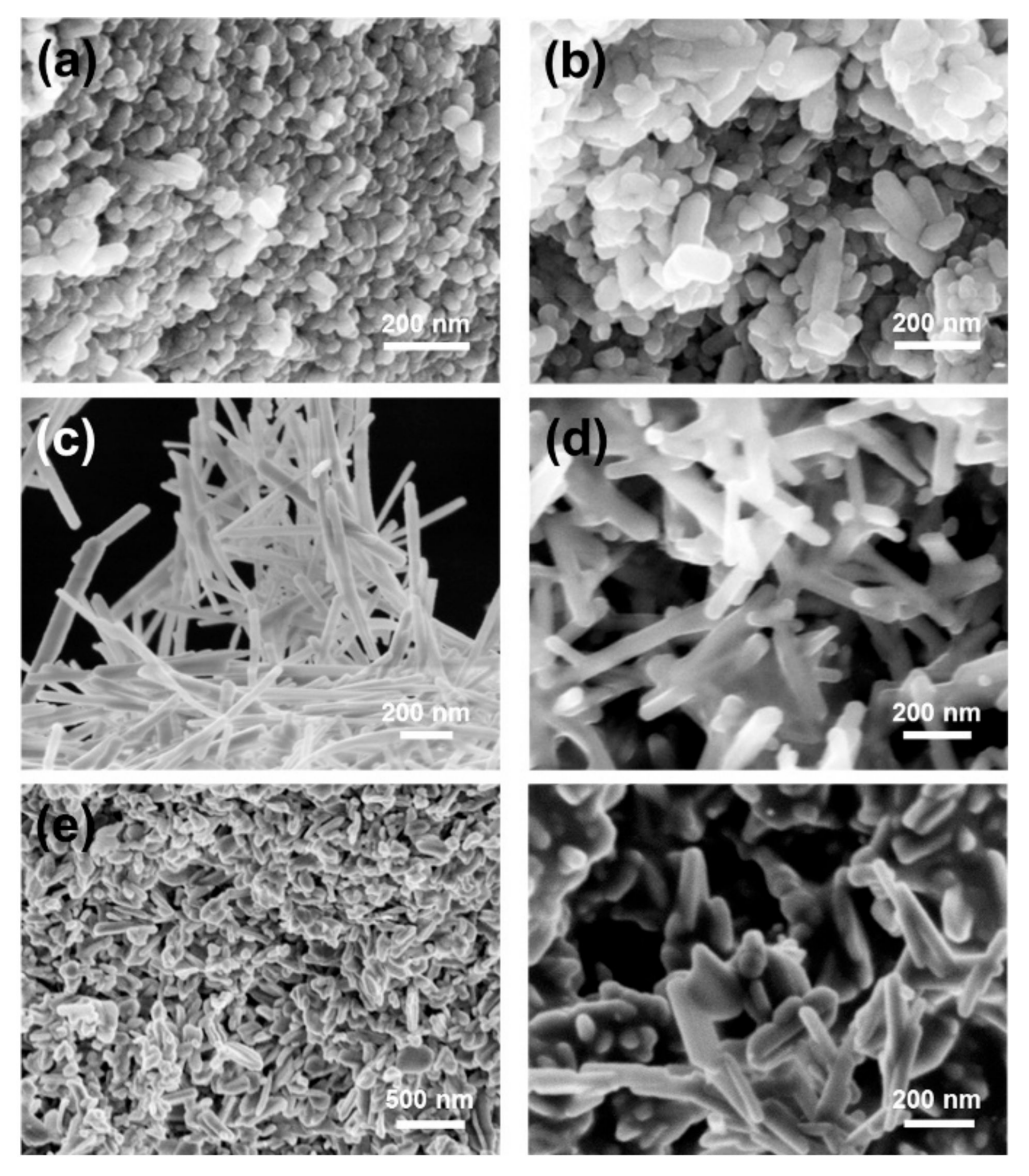
Different morphologies of HAP obtained from the methods proposed by Sans et al. [75]: (**a**) spherical polymorphs; (**b**) rod shapes; (**c**) belts; (**d**) belts with smaller aspect ratio; (**e**) low magnification of needle-flakes; and high magnification of needle-flakes. With permission from ACS Publications. https://doi.org/10.1021/acs.cgd.0c00850 (accessed on 5 April 2021).

In a recent work the effect of different matrix components on the steps and processes taking place in the formation of HAP from an amorphous calcium phosphate (ACP) precursor phase was investigated. In the absence of additives, the process starts with the formation of ion pairs that subsequently lead to the appearance of an ACP phase that in the last step is transformed into HAP. In the presence of alginate-based additives, the formation and lifetime of the ACP phase can be modified due to the stabilization of the intermediate pairs. In the presence of guluronate-unit (G-unit) additives, the final precipitates were composed of a mixture of octacalcium phosphate and HAP. The work helps to understand the complex biomineralization processes in the presence of multiple components [74]. Sans et al. [75] developed a robust method based on hydrothermal methods but without using any additive, only using organic solvent to produces pure and crystalline HAP with a controlled shape and size. Here, ethanol has been proven to allow the control of the crystals’ morphology (spherical polymorphs, rods, and belts, see Figure 4) through pH and their sizes through ion concentration. The formation of needle flake-like crystals and micrometric rods was achieved with different solvents and pH.

## 4. Application of Modern Computational Methods in HAP Based Materials

Nowadays, the advance of computational tools has already outstripped their original settlement in the purest scientific disciplines to address any area [76,77,78,79]. Many topics remained elusive due to their complexity, but the continuous and unison advance in hardware and software has managed to remedy this. The main goal of these methods is to predict the nature and strength of interactions, to improve relevant biological data, to better understand all the factors involved in the occurrence of a given response, etc. [80,81]. Computational techniques involve characterization across multiple scales and levels of organization. In general, the discovery of new materials is very challenging, costly, and time-consuming. In this sense, advanced computational tools, and methods such as: QSAR, combinatorial chemistry, machine learning, molecular dynamics and molecular docking techniques have become powerful tools at various stages of materials and process development [82,83].

There are hardly any publications where machine learning algorithms are applied to hydroxyapatite synthesis processes; the novelty of the method and the scarcity of databases are the main reasons for this. However, this panorama will undoubtedly change soon. For these reasons, we consider it of vital importance to comment on some of the scarce contributions.

Manufacturing processes are widely used for the fabrication of ceramic, composite and polymer materials. Selective laser sintering is one of them which used a laser beam to selectively fuse the powder into a designed solid object layer by layer. Garg et al. [84], trying to predict the porosity of HAP, applied a multi-gene genetic algorithm based on the statistical approach of stepwise regression and classification methods: Support Vector Machines (SVM), Bayesian classifier and Artificial Neural Networks (ANN). Thus, the desired open porosity values can be achieved by regulating the values of the three input variables: laser scan speed, layer thickness and laser power. Although some important parameters such as energy efficiency or thermal cracking were not considered in the model, the results showed good agreement with experimental studies.

Early studies have confirmed the role of functional groups in early nucleation of HAP by means of their transmittances at different wavelengths. In order to be able to predict a priori the growth capacity of HAP materials, Okafor [85] reported the use of two ensemble learning methods, Random Forest and Extreme Gradient Boosting, for predicting the transmittance levels for a given wavenumber of infrared radiation through the bulk of HAP specimens. The predictions were tested with experimental results showing an excellent agreement and a very good generalization for predicting the transmittance levels.

One of the most common processes to improve certain characteristics of hydroxyapatite is to dope it with ions (Na^+^, K^+^, Ag^+^, Ni^2+^, Mg^2+^, Sr^2+^, Zn^2+^, Ce^3+^, …). For this reason, Yu et al. [86] have performed an experimental and computational study to explore and optimize mechanosynthesis and structural features modeling of the substituted hydroxyapatite. The employed machine learning techniques were ANN with a multilayer perceptron architecture (MLP) and Genetic Programming (GP). The values for input variables were the chemical compositions of the reagents. The outputs were three structural characteristics: crystallite size, microstrain and grain boundary volume fraction. The models were able to predict with good accuracy the structural characteristics of the materials based on their chemical constituents, which is a huge time and cost saver.

In order to analyze the binding process of proteins on hydroxyapatite, a model based on Quantitative Structure Property Relationship (QSPR) was developed. The dataset was constructed with experimental data (chromatography) and theoretical molecular descriptors. Finally, non-linear SVM algorithms were successfully trained and tested. Thus, the model is a good predictive tool for the prediction of protein-HAP affinities [87].

Thinking more about applications outside the field of health, one group has highlighted the importance of static dielectric constant in electric and optical properties of HAP by using density functional theory (DFT), Vienna Ab initio Simulation Package (VASP, a computer program for atomic scale materials modelling) within the Kohn-Sham formalism [88]. The same computational modelling was successfully applied to investigate the energetics of the HAP surfaces [89], to predict physical and magnetic properties of different ion-substituted HAP powders [90], to discuss the role of impurities in HAP in the uptake and release of divalent metal ions during mineralization [91] or to report the temperature-dependent partition functions and unit cell parameters for the HAP molecule [92]. All these studies demonstrate the versatility of the method in relating intrinsic properties at the atomic or energy band level.

At another time or dimensional scale, methods based on first principles are not applicable, being necessary to resort to other methods such as numerical methods or finite element analysis.

Thus, Palazzo et al. [93] performed a numerical approach, based on the use of the Finite Element Method to describe the drug release kinetics from the porous hydroxyapatite matrix with an excellent agreement with experimental data. The authors developed several models based on the assumption that diffusion is equal in both porous matrix and solvent, disregarding the convective contribution to drug transport and solving the species concentration equation:

(1) $$\frac{\partial c}{\partial t}=div\left(D\;grad\;c\right)+q_{c}=\nabla^{2}c+q_{c}$$

where *c* is the species mass concentration, *D* is the mass diffusion coefficient for drug in the domain in which the equation is solved and *q_c_* is a sink/source term. They found that the less porous the ceramic graft is, the more evident is the initial burst release. This behavior can be due to the trend for the drug molecules to concentrate themselves on the external macropore walls. The trend becomes more evident with the decreasing in the ceramic porosity and surface area, because of the difficulty for the hydrocortisone to reach the internal micropores. The same group demonstrates that HAP nanocrystals and antitumor drugs can be conjugated in such a way as to yield a smart bone filler delivery system, acting both as bone substitutes and as platinum drug releasing agents with the final goal of locally inhibiting the tumor re-growth and reducing the systemic toxicity. The one here described not only can ensure a prolonged release of active species but also improves the performance of the unmodified drug. Moreover, these results suggest the possibility of using the chemicophysical differences of HAP nanocrystals, above all degree of crystallinity, crystal size and surface area, in order to strongly tailor the Pt complex release kinetics [94].

Finite element analysis is becoming increasingly important in microfluidic device design that allows scientists to explore different microfluidic device designs to optimize and automate different chemical processes and applications. Obviously, the HAP synthesis and formulations processes could not remain on the sidelines of these advances, and as a sample of them, we would like to highlight some of the most recent contributions.

**Figure 5:**
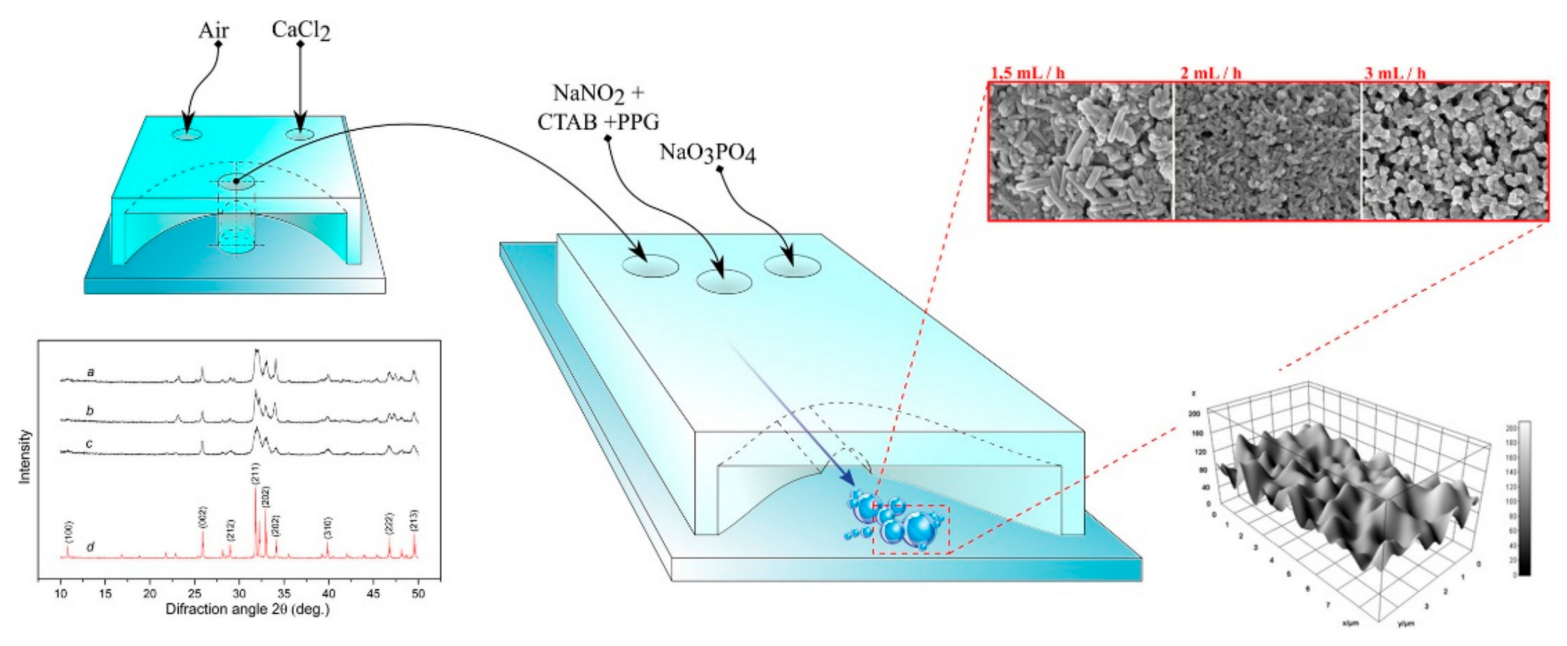
Schematic of the microfluidic device used for HAP nanoparticles production. The SEM images show the different materials obtained at different flow rates, as well as the surface roughness. With permission from Elsevier. https://doi.org/10.1016/j.msec.2019.04.037 (accessed on 8 April 2021).

In a recent work, we have theoretically and experimentally proposed a noble system for the synthesis of hydroxyapatite nanoparticles using a microfluidic device realized by continuous laminar flow (see Figure 5). The materials showed the same properties as those produced by conventional methods; however, the device is able to design nanoparticles of different size by varying the flow. This holds potential for optimizing various processes for the synthesis of nanostructures and beyond, making it very cost-effective economically [95].

In the same line as the preceding work, another microfluidic device was designed to produce rod-shaped HAP nanoparticles with an adjustable size of 55–95 nm. The average size of the nanoparticles was also adjustable as a function of flow rate. The relationship obtained between nanoparticle size and inner tube geometry was interesting [96]. In another interesting study, microfluidics was used to design a bone microenvironment composed of hydroxyapatite and fibrin to investigate the interactions that take place. For example, increasing the concentration of HAP affects cell viability and proliferation of cancer cells. We also showed that cell migration was inversely proportional to HAP concentration [97]. Along the same lines, the bibliography offers original studies covering different objectives that are worth consulting [98,99,100,101,102,103,104,105,106,107].

## 5. Applications of Smart Hydroxyapatite-Based Materials

Smart materials are those that show an observable effect in one of their facets when stimulated from another. In this way, different facets can be covered such as mechanical, electrical, chemical, optical, thermal, etc. The purpose of these materials is to provide new capabilities or improve the performance of existing ones through an agile and flexible design. Obviously, a material as versatile as hydroxyapatite has not remained unaffected by this new trend. Thus, by means of different processes, many researchers have made it possible to broaden the initial concepts to design new structures that can monitor their structural integrity or can adapt to the environment. The following are some of what we consider to be the most relevant ones.

**Figure 6:**
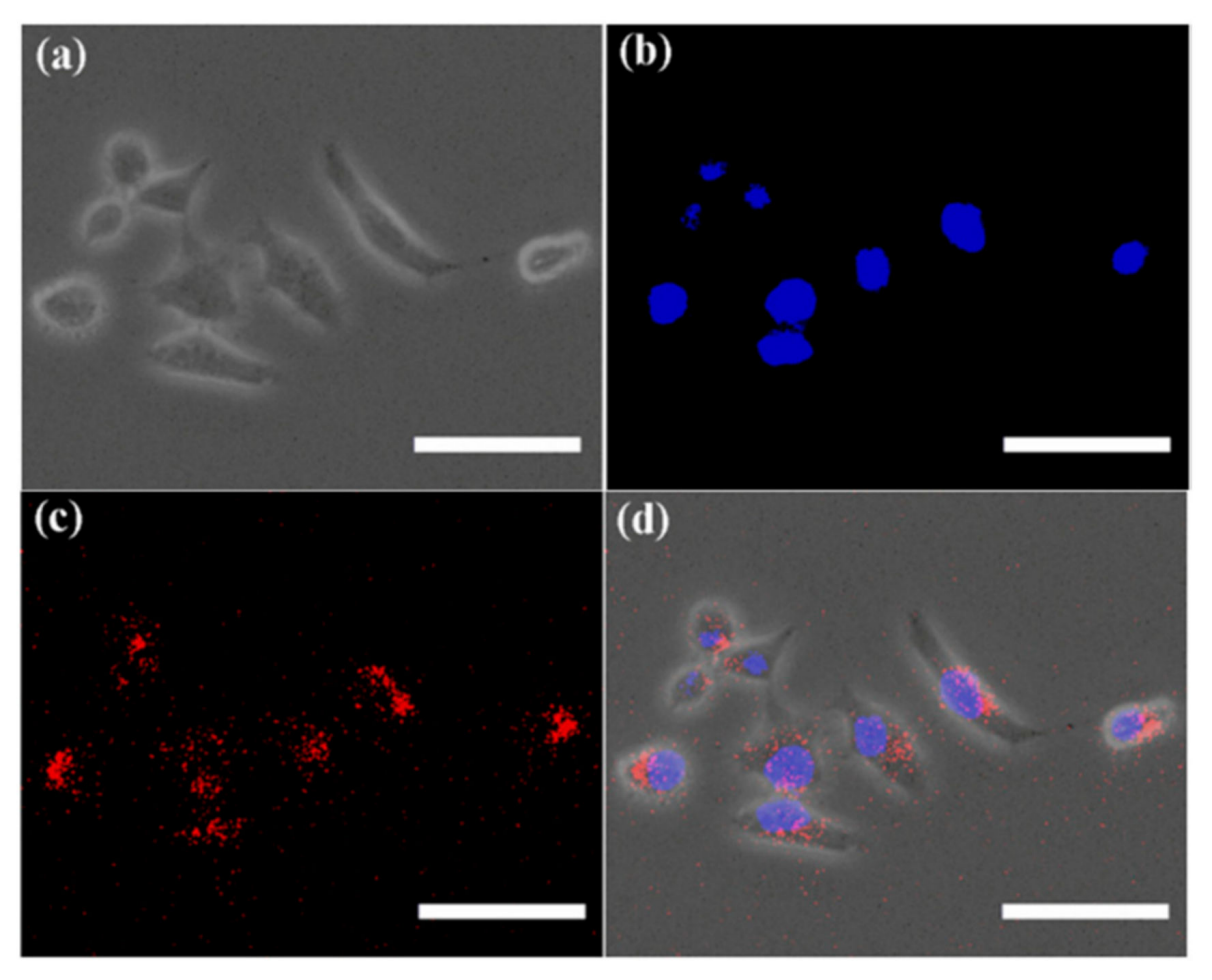
Confocal Laser Microscopy images of cancer cells co-cultured with sodium heparin coated Eu^3+^ HAP. (**a**) bright field; (**b**) blue: cell nuclei (**c**) red fluorescence observed in the cytoplasm of cells around cell nuclei, demonstrating the feasibility of SH-Eu:nHAP as bioimaging agent; (**d**) merged image. Scale bars are 50 μm. With permission from Elsevier. https://doi.org/10.1016/j.colcom.2019.01.001 (accessed on 19 May 2021).

Methods aimed at the design and development of nano-sized HAP and the study of their properties at the nanoscale have been one of the first steps in the development of hydroxyapatite-based smart materials, being used for colloid stability [108], drug delivery [109] or gene therapy [110,111]. In a previous study, we [112] developed a new route, based on the method proposed by Liu [14], that involves surfactants and block copolymers organized networks to create bioactive superstructures. The method lets us obtain bioactive and biocompatible materials which replicate the bone structure: HAP nanorods of 25–50 nm length organized in hierarchical structures. The variation of the compounds allowed us to modify the chemical and/or the surface properties on the materials. The nature of the polymer alters the distance between HAP nanorods and consequently, micro rough and porosity. Materials surface was revealed as a key factor in their bioactivity properties: on rough surfaces, the valleys seem to be the preferential sites for the nucleation and growth of calcium phosphates. Thus, the acceleration of the nucleation of the apatite over the materials, bioactivity and morphology is attributed to the surface topographical changes at the micrometer level. In another study, we designed a new material based on gelatin nano-HAP and tannic acid [113]. Tannic acid was chosen for two reasons: on the one hand, it has antioxidant, antimicrobial and cardioprotective properties. It also acts as a cross-linking agent to reduce the solubility of gelatin at human body temperature. The results showed that the presence of nano HAP in gelatin scaffolds exerts a great impact on the biomineralization process and scaffold strength enhancement. The crosslinking effect of TA has hardly any effect on the structure but generates a significant increase in the thermal stability of the scaffold. Ion doping has been one of the most frequently used modifications to try to improve the essential physicochemical properties that lead to specific biological behavior after implantation [114]. For example, the addition of a naturally important cation, such as Mg^2+^, into the nano HAP structures resulted in the formation of calcium-deficient HAP, enhanced cell viability, propagation and proliferation in vitro, and improved osteoblast adhesion [115]. Molybdenum oxides were also employed in doped HAP. Thus, through a delicate control of the reaction conditions, a biocompatible material that simultaneously exhibited blue and red self-activated fluorescence at room temperature and antibacterial activity was obtained [116]. In relation to the antibacterial properties, a material composed by HAP, alginate and silver nanoparticles was successfully developed [117]. Furthermore, in bioimaging applications, the capacity and solvency of hydroxyapatite was demonstrated through its doping with Eu^3+^, showing good biological safety and characteristic luminescence emission [118], Figure 6.

Layer by layer, LbL, is a straightforward and versatile bottom-up surface modification technique that can be applied to coat substrates with different geometries, from simple planar membranes to complex three-dimensional shapes. Its main advantage is the possibility for the incorporation of several classes of materials, including biomolecules and inorganic particles. In this sense, LbL allows the modulation of the distribution of different composites for applications such as biomaterial interfaces for implantable systems intended for bone regeneration [119,120].

Proteins are the most versatile biological entities, performing structural, enzymatic, defense or transport functions [121,122]. Given their versatility, it was inevitable to characterize their interactions with a material with multiple applications in medicine such as hydroxyapatite. Serum albumin is the most abundant human body extracellular protein, the major calcium-binding protein and it is known to be the first to surround foreign bodies when they are in contact with blood. For these reasons, the interaction of the nano-HAP with albumin was analyzed in order to check the effect of the protein in the biomineralization process [123]. The results showed that albumin has a dual role in HAP mineralization: on the one hand it coats the active sites on the mineral surface by slowing down ionic diffusion; on the other hand, it facilitates the dissolution of calcium ions by reducing the formation of new calcium phosphate layers. The kinetics of the interaction between albumin and HAP was also characterized, the results showed that it is a slow process where albumin adapts perfectly to the HAP surface, forming a hard protein corona consisting of a single layer [124]. The effect of HAP on the mechanical properties of different scaffolds could be characterized by a combination of rheological measurements and appropriate viscoelastic models [125,126].

The need to prevent implant-derived infections or inflammation has highlighted the need to use drugs during treatment. An effective way is to include the drugs inside the implant itself, thus achieving an adequate dosage, avoiding aggregates [127,128,129], and in situ release. Hydroxyapatite has been shown to be an efficient drug delivery vehicle as demonstrated by multiple publications [130,131]. A recent work details the adsorption and release of two drugs onto nano HAP [132]. The adsorption and release mechanisms were clearly stated by using different theoretical models. The Sips model best reproduced the experimental results for adsorption, while for release, the best model was the Korsmeyer-Peppas one.

Catalysis is crucial for the chemical industry, and hydroxyapatite, as a basic material, has not been left behind in this field. In fact, as a catalyst, HAP has the unusual property of containing both acid sites and base sites in a single-crystal lattice. Examples of hydroxyapatite-based materials for catalysis are numerous. In order to exceed the theoretical yield of 50% in asymmetric catalysis for obtaining enantiomerically pure compounds, Ru3+ ions were immobilized on hydroxyapatite by an ion-exchange procedure, the ratios were optimized to obtain the maximal catalytic performance for secondary alcohol racemization [133]. Tsuchida et al. [134] developed a process for synthesizing biogasoline from bioethanol, in one step, with high selectivity, over a highly active nonstoichiometric HAP catalyst. The removal of NO and NO_2_ produced by diesel and gasoline engines is a key issue affecting the environment and the automotive industry. One of the solutions is based on the use of active and stable catalysts for the elimination of these products. Again, hydroxyapatite, due to its high adsorption capacity and surface acidic/basic properties, has attracted much attention as support for transition metal catalysts in various reactions. Thus, Copper-loaded hydroxyapatite prepared by ion-exchange has been found to be active, and selective in the NH_3_ selective catalytic reduction in NO and NO_2_ by NH_3_ [135]. Gold catalysts have been applied in a variety of important reactions, including CO oxidation, water–gas shift and the water pollutant removal. However, most gold catalysts still suffer from the easy sintering of gold particles at elevated temperature and poor long-term stability, which is the major obstacle to the practical application of gold catalysts. For this reason, a highly efficient and stable Au–Cu/hydroxyapatite catalyst, prepared through a deposition–precipitation method, was proposed, where, due to the synergistic effect between gold and CuOx in the hydroxyapatite support, the Au–Cu/HAP catalyst showed superior activity and stability [136]. By modulating the surface area and porosity, functional hydroxyapatite devices for nanofiltration and catalysis can be designed. Webler et al. [137] achieved surface areas above 200 m^2^g^−1^ and a degree of porosity close to 70% by using latex bead templates. Chen et al. [138] were the first in utilizing the animal bone to design and prepare cost-effective and stable catalytic systems by using wet impregnation method for biodiesel production. Once loaded with K_2_CO_3_ on the pig bone, the materials exhibit significantly enhanced catalytic activity. The authors evaluate different methods changing calcination temperature and K_2_CO_3_ loading. The best result, biodiesel yield 96.4%, was found with a 30% loading and a temperature of 600 K.

Filtration is very important for many industrial process and water reuse treatment. The ability of hydroxyapatite to successfully remove ions from aqueous solution makes it an ideal candidate for many filtration processes [139]. Inoue et al., by using a high magnetic field, managed to orient the crystalline axes of the hydroxyapatite, knowing that proteins adsorb selectively on the different planes of hydroxyapatite, they were able to develop a selective filter that allows the separation of different types of proteins [140].

A highly flexible membrane based on TiO_2_-coated HAP whiskers was developed, which, by optimizing the electrostatic interactions between the TiO_2_-coated HAP whiskers and nanoparticles, allowed the separation of gold nanoparticles with a filtration efficiency close to 100% [141].

**Figure 7:**
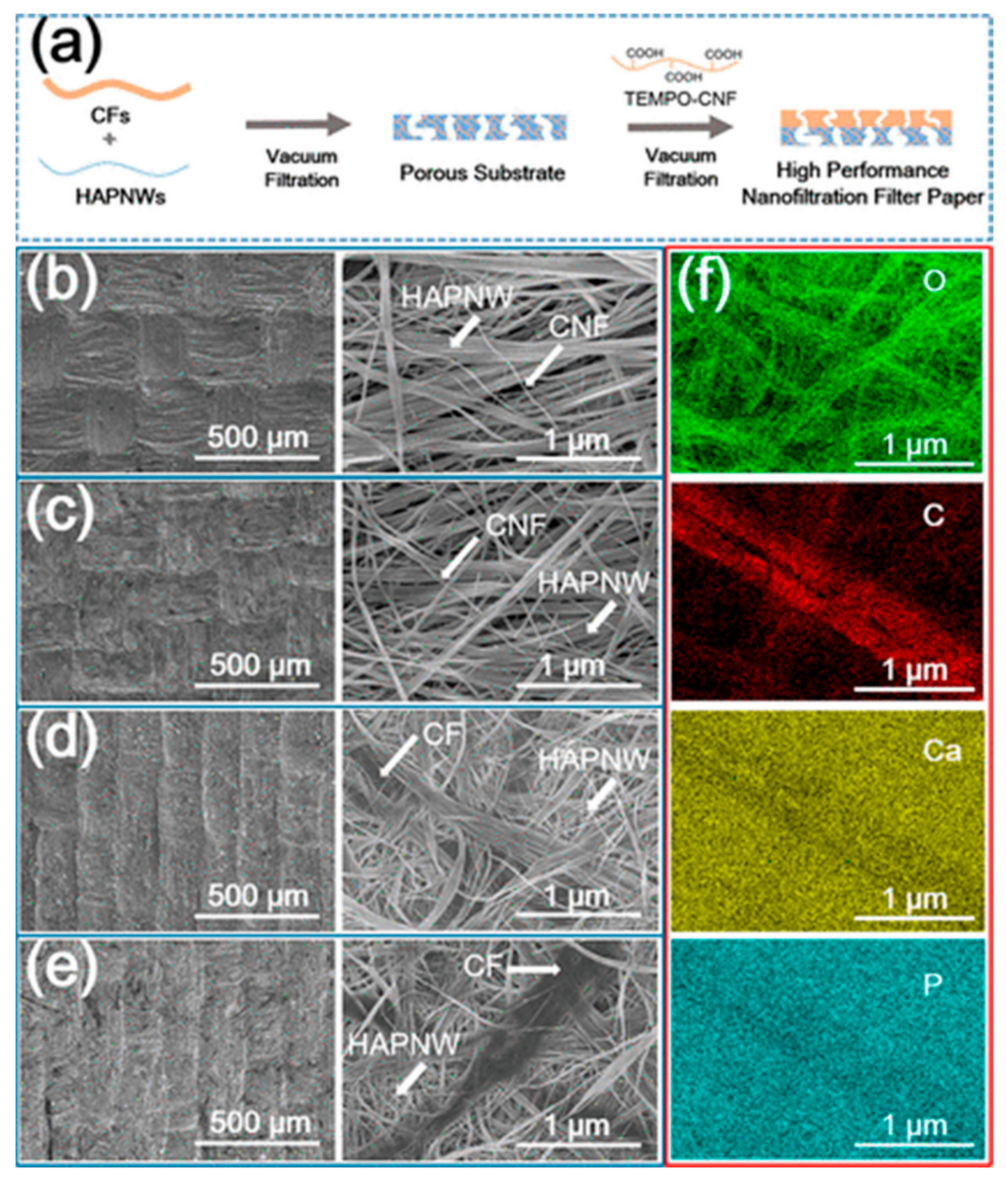
(**a**) Schematic illustration for the preparation process of the membrane: CFs (cellulose fibers), HAPNWs (ultralong HAP nanowires). (**b**–**e**) SEM images of the memebrane with different cellulose weight ratios: (**b**) 20 wt% CNFs; (**c**) 40 wt% CNFs; (**d**) 20 wt% CFs; (**e**) 40 wt% CFs. (**f**) Energy dispersive spectroscopy mappings of the filter with 20 wt% CFs., showing the distribution of O, C, CA and P elements in the filter. With permission from ACS Publications. https://doi.org/10.1021/acssuschemeng.9b03793 (accessed on 14 April 2021).

Nanofiltration membranes thanks to their low operation pressure, high flux, and high retention of multivalent anion salts, can remove different contaminants from water. One of the most common technologies to manufacture them has been electrospinning. However, the membranes manufactured in this way are usually thin layers, the preparation time is relatively long, and it is complicated to regulate the structure. To solve these problems, Zhang et al. [142] developed a novel kind of nanofiltration filter paper based on cellulose nanofibers on a porous filter paper consisting of ultralong HAP nanowires and cellulose fibers, see Figure 7. The pure water flux of the novel membrane is about 50 times that of the pure filter paper. Besides, compared with electrospun membranes, the new route saves times and it is ecofriendly, making these new membranes a much more sustainable solution.

Other interesting examples include the filtering of microbes [143], virus [144], phosphorus [145], particulate matter (PM) [146], soil pollution [147].

The inclusion of magnetic particles in hydroxyapatite scaffolds provides new and interesting features, especially the detection and control of the scaffold by non-invasive methods. Within the field of medicine, it allows the visualization of in vivo applications by means of magnetic resonance imaging; it stimulates cell adhesion, proliferation, and differentiation; it allows the accumulation of growth factors, drugs and so on. For these reasons, many researchers have focused their efforts in this field [148,149]. With respect to the structural properties of these complexes, the degree of fusion between the nanoparticles and the matrix can be very high, reaching the point of exchanging atoms, for example electron spin resonance measurements indicate that the Co^2+^ ions were substituted into the Ca) sites in HAP lattice and X-ray diffraction shown the substitution of Fe^3+^ ions into the HAP matrix [150]. With antibacterial properties in mind, Fe_3_O_4_/HAP nanoparticles have been synthesized by a homogeneous precipitation method. The study discovered that calcination temperature influences the magnetic and photocatalytic activity of the material, the optimal calcination temperature being 400 °C [151]. Application of nanoparticles as heat carriers remains subject to limited biocompatibility because they must be introduced into the blood vessels. To overcome this problem, nanoparticles have been coated to date with biocompatible materials, in this sense, HAP-coated ferrite nanoparticles are the most promising material. Inukai et al. proposed a novel route using a two-step procedure. First, the ferrite particles were synthesized by co-precipitation. Second, the suspension, plus Ca(NO_3_)_2_ and H_3_PO_4_, was nebulized into mist ultrasonically. Then the mist was pyrolyzed at 1000 °C to synthesize HAP–ferrite hybrid particles [152]. To conclude this section, we could not forget all the work that Anna Tampieri and her group have done on this subject, so we recommend to all those interested in this particular field to consult her proficient bibliography [153,154,155,156,157,158,159,160,161,162,163].

## 6. Conclusions

In a review as comprehensive as the one presented here, a perspective on possible future developments seems timely. One might intuitively sense that the trend of the references in this review already gives the reader an idea of possible directions. The development of new hydroxyapatite-based smart materials often results in advanced materials with attractive morphological, mechanical, and biological properties and enhanced performance. In some cases, these new routes confer to scaffolds unique properties such as strength improvements, limitation of postoperative complications by improving range of motion or decreasing pain. From an experimental point of view, the choice of techniques is fundamental for the processing of materials in terms of performance, activity and even reducing time and economic costs and improving environmental sustainability. From a theoretical point of view, the formulation of a realistic model to describe the interactions between all the constituent elements, the experimental parameters and the functionality of the material is still to be developed. It is in this area that modeling can provide valuable insights in the near future. A greater interrelation between experimental and theoretical approaches is expected, which will undoubtedly generate a positive synergy. Thus, the use of HAP in customized tissue regeneration systems, biphasic coatings, smart drug delivery systems, purification, or energy, both on a personal and industrial scale are viable and very promising options in this area for the future.

## Sample Availability

Samples of the compounds are available from the authors.
